# *catena*-poly[[[(2,2′-bipyridine-2κ^2^ *N*,*N*′)-μ-cyanido-1:2κ^2^ *N*:*C*-cyanido-2κ*C*-tris­(methanol-1κ*O*)(nitrato-1κ^2^ *O*,*O*′)iron(II)yttrium(III)]-di-μ-cyanido-1:2′κ^2^ *N*:*C*;2:1′κ^2^ *C*:*N*] methanol solvate hemihydrate]

**DOI:** 10.1107/S1600536810029843

**Published:** 2010-07-31

**Authors:** Yan Xu, He-Qing Shu, Xiao-Ping Shen

**Affiliations:** a Suqian College, Suqian 223800, People’s Republic of China; b School of Chemistry and Chemical Engineering, Jiangsu University, Zhenjiang 212013, People’s Republic of China

## Abstract

The title complex, {[Fe^II^Y^III^(CN)_4_(NO_3_)(C_10_H_8_N_2_)(CH_3_OH)_3_]·CH_3_OH·0.5H_2_O}_*n*_, is built up of ladder-like chains oriented along the *c* axis. Each ladder consists of two strands based on alternating Fe^II^ and Y^III^ ions connected by cyanide bridges. Two such parallel chains are connected by additional cyanide anions (the ‘rungs’ of the ladder), which likewise connect Fe^II^ and Y^III^ ions, such that each [Fe(bipy)(CN)_4_]^2−^ (bipy is 2,2′-bipyridine) unit coordinates with three Y^III^ ions and each Y^III^ ion connects with three different [Fe(bipy)(CN)_4_]^2−^ units. The Fe^II^ atom is six-coordinated in a distorted octa­hedral geometry and the Y^III^ atom cation is eight-coordinated in a distorted dodeca­hedral environment. The uncoordinated methanol solvent mol­ecules are involved in hydrogen-bonding inter­actions with the one terminal cyanide group and a coordinated methanol mol­ecule from another [Y^III^(NO_3_)(CH_3_OH)_3_]^2+^ unit. Adjacent ladder-like chains are also held together by hydrogen bonds between the terminal cyanide ligands of the [Fe(CN)_4_(bipy)]^2−^ units in one chain and the OH donors of CH_3_OH ligands from [Y^III^(NO_3_)(CH_3_OH)^3^] units in neighboring chains. The water molecule exhibits half-occupation.

## Related literature

For background to the design, synthesis and properties of mixed rare earth–transition metal complexes, see: Wilson *et al.* (2009 ▶); Zhou *et al.* (2002 ▶); Li *et al.* (2008 ▶); Karan *et al.* (2002 ▶); Sokol *et al.* (2002 ▶); Toma *et al.* (2003 ▶); Xu *et al.* (2009 ▶). For related structures, see: Baca *et al.* (2007 ▶); Liu *et al.* (2008 ▶); Yuan *et al.* (2004 ▶).
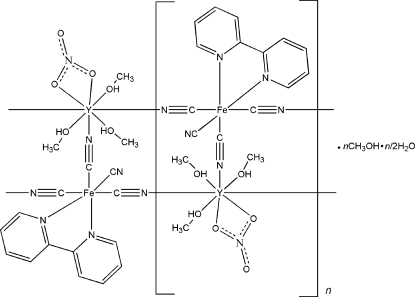


## Experimental

### 

#### Crystal data

[FeY(CN)_4_(NO_3_)(C_10_H_8_N_2_)(CH_4_O)_3_]·CH_4_O·0.5H_2_O
*M* *_r_* = 604.21
Monoclinic, 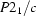

*a* = 12.803 (3) Å
*b* = 18.132 (4) Å
*c* = 10.728 (2) Å
β = 103.439 (3)°
*V* = 2422.2 (9) Å^3^
*Z* = 4
Mo *K*α radiation
μ = 3.04 mm^−1^
*T* = 173 K
0.26 × 0.22 × 0.20 mm

#### Data collection

Bruker SMART APEX CCD diffractometer
Absorption correction: multi-scan (*SADABS*; Bruker, 2002 ▶) *T*_min_ = 0.46, *T*_max_ = 0.55
18891 measured reflections
4760 independent reflections
3490 reflections with *I* > 2σ(*I*)
*R*_int_ = 0.067

#### Refinement

*R*[*F* ^2^ > 2σ(*F* ^2^)] = 0.052
*wR*(*F* ^2^) = 0.107
*S* = 1.07
4760 reflections
316 parameters
H-atom parameters constrained
Δρ_max_ = 0.49 e Å^−3^
Δρ_min_ = −0.57 e Å^−3^

### 

Data collection: *SMART* (Bruker, 2004 ▶); cell refinement: *SAINT* (Bruker, 2004 ▶); data reduction: *SAINT*; program(s) used to solve structure: *SHELXS97* (Sheldrick, 2008 ▶); program(s) used to refine structure: *SHELXL97* (Sheldrick, 2008 ▶); molecular graphics: *SHELXTL* (Sheldrick, 2008 ▶) and *DIAMOND* (Brandenburg, 2006 ▶); software used to prepare material for publication: *SHELXTL*.

## Supplementary Material

Crystal structure: contains datablocks I, global. DOI: 10.1107/S1600536810029843/nc2192sup1.cif

Structure factors: contains datablocks I. DOI: 10.1107/S1600536810029843/nc2192Isup2.hkl

Additional supplementary materials:  crystallographic information; 3D view; checkCIF report

## Figures and Tables

**Table 1:** Hydrogen-bond geometry (Å, °)

*D*—H⋯*A*	*D*—H	H⋯*A*	*D*⋯*A*	*D*—H⋯*A*
O5—H5*A*⋯N6^i^	0.99	1.78	2.762 (5)	172
O7—H7*B*⋯N6	0.85	2.03	2.823 (5)	154

Symmetry code: (i) 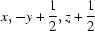.

**Fig. 1.**
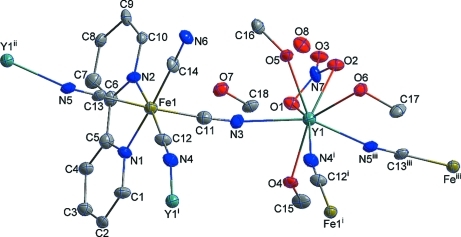
The molecular structure of the title complex, showing the atom-numbering scheme. Displacement ellipsoids are drawn at the 30% probability level. [Symmetry codes: (i) -x + 1, -y, -z + 1; (ii) x, -y, z - 1; (iii) x, y, z + 1.]

**Fig. 2.**
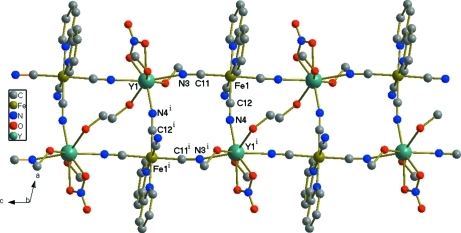
The one-dimensional chain of the title complex. [Symmetry code: (i) -x + 1, -y, -z + 1.]

## supplementary crystallographic information

### Comment

Much attention is currently devoted to the design and synthesis of mixed rare earth–transition metal complexes because rare earth ions have a rich coordination chemistry with high coordination numbers and significant coordination flexibility, which often leads to unanticipated but remarkable structures (Karan *et al.*, 2002; Li *et al.*, 2008; Wilson *et al.*, 2009; Zhou *et al.*, 2002.) [*M*(CN)*_x_*(*L*)*_y_*]*^n-^* [*M* = Cr, Fe, Ru and Mo; *L* = chelate ligand; and *x* = 2, 3, 4) can be used as the bricks to synthesize low-dimensional cyanide-bridged bimetallic compounds, which is a elaborate strategy as revealed by a few research groups (Sokol *et al.*, 2002; Toma *et al.*, 2003). However, the assemblies of [*M*(CN)*_x_*(*L*)*_y_*]^n-^ with rare earth ions have rarely been reported so far (Xu *et al.*, 2009). In this paper, we report a new cyano-bridged Fe^II^Y^III^ bimetallic ladder-like chain complex, based on the [Fe^II^(bipy)(CN)_4_]^2-^ [bipy = 2,2'-bipyridine] building block.

The asymmetric unit in the structure of the title complex comprises one [Fe^II^(bipy)(CN)_4_]^2-^ anion, one [Y^III^(NO_3_)(CH_3_OH)_3_]^2+^ cation, one solvent methanol molecule and half a water molecule (Fig. 1). The crystal structure consists of one-dimensional ladder-like bimetallic chains, {[Fe^II^(bipy)(CN)_4_][Y^III^(NO_3_)(CH_3_OH)_3_]}*_n_*, built up from alternating Fe^II^ and Y^III^ metal centers linked through the cyano bridges (Fig. 2). The ladder-like bimetallic chains contain Fe_2_Y_2_ centrosymmetric motifs. The [Fe(bipy)(CN)_4_]^2-^ fragment exhibits a distorted octahedral structure consisting of two N atoms from a planar bipy ligand and four C atoms from four CN^-^ groups. The small bite angle subtended by the chelating bipy group [79.90 (15)° for N1—Fe1—N2] is one of the main factors accounting for this distortion. Three of the four cyano groups of the [Fe(bipy)(CN)_4_]^2-^ unit are bridging, while the fourth is terminal. The Fe—C—N angles for both terminal [178.6 (5)°] and bridging [178.7 (4), 179.4 (4) and 174.4 (4)°] CN^-^ groups deviate slightly from strict linearity. Each Y^III^ cation is eight-coordinated, connecting with two O atoms from the NO_3_ group, three O atoms from three CH_3_OH units and three N atoms from three CN^-^ ligands, building distorted YN_3_O_5_ dodecahedral surroundings (Fig. 1). The Y—O bond lengths fall in a very narrow range [2.385 (3)–2.448 (3)Å for Y—O(NO_3_) and 2.372 (3)–2.392 (3)(3) Å for Y—O(CH_3_OH)]. The Y—N(cyanide) bond distances [2.401 (4)–2.344 (4) Å] are somewhat smaller than those from {[Ru(phen)(CN)_4_]_3_[Ln(terpy)(H_2_O)_3_]_2_.*n*H_2_O}_∞_ [2.530 (9)–2.548 (11) Å; Baca *et al.*, 2007]. The angles of *Y*^III^—*N*—*C*(cyano) are far from linear [165.9 (3)–169.9 (3)°]. The NO_3_^-^ ion acts as a bidentate ligand toward Y^III^ through two of its three O atoms, which is different from previously reported cases (Yuan *et al.*, 2004; Liu *et al.*, 2008), in which an NO_3_^-^ ion coordinated to a rare earth ion acts as a monodenate ligand in rare earth–transition metal complexes.

The Fe···Y separations across cyanide bridges are 5.410 (4), 5.357 (3) and 5.424 (4) Å. The uncoordinated methanol solvent molecules are involved in hydrogen-bonding interactions with the one terminal cyanide group and a coordinated methanol molecule from another [Y^III^(NO_3_)(CH_3_OH)_3_]^2-^ unit (Table 1). Adjacent ladder-like chains are also held together by hydrogen bonds between the terminal cyanide ligands of the [Fe(CN)_4_(bipy)]^2-^ units in one chain and the OH donors of CH_3_OH ligands from [Y^III^(NO_3_)(CH_3_OH)^3^] units in neighboring chains. From this arrangement a two-dimensional structure is formed.

### Experimental

Red brown prism crystals of the title complex were obtained by slow diffusion of a MeOH solution of K_2_[Fe(bipy)(CN)_4_].3H_2_O(0.1 mmol) and an aqueous solution of Y(NO_3_)_3_.6H_2_O (0.1 mmol) through an H-tube at room temperature. The resulting crystals were collected, washed with H_2_O and MeOH, respectively, and dried in air.

### Refinement

The (C)H atoms of the bipy ligand were placed in calculated positions (C - H = 0.95 Å) and refined using a riding model, with *U*_iso_(H) = 1.2*U*_eq_(C). The (C)H atoms of the methanol molecule were placed geometrically (C - H = 0.98 or 0.96 Å) and refined as riding, with *U*_iso_(H) = 1.5*U*_eq_(C). The (O)H atoms of the methanol molecule were located in a difference Fourier map and refined with O - H restraints (O - H = 0.99 or 0.85 Å), and with *U*_iso_(H) = 1.5*U*_eq_(O). The position of the water molecule is occupied to only 50%. Its H atoms were located in a difference Fourier map and refined with O - H restraints (O - H = 0.85 Å), and with *U*_iso_(H) = 1.5*U*_eq_(O).

### Crystal data

**Table:**

[FeY(CN)_4_(NO_3_)(C_10_H_8_N_2_)(CH_4_O)_3_]·CH_4_O·0.5H_2_O	*F*(000) = 1228
*M**_r_* = 604.21	*D*_x_ = 1.657 Mg m^−^^3^
Monoclinic, *P*2_1_/*c*	Mo *K*α radiation, λ = 0.71073 Å
Hall symbol: -P 2ybc	Cell parameters from 838 reflections
*a* = 12.803 (3) Å	θ = 3.0–23.5°
*b* = 18.132 (4) Å	µ = 3.04 mm^−^^1^
*c* = 10.728 (2) Å	*T* = 173 K
β = 103.439 (3)°	Prism, red brown
*V* = 2422.2 (9) Å^3^	0.26 × 0.22 × 0.20 mm
*Z* = 4	

### Data collection

**Table:**

Bruker SMART APEX CCD diffractometer	4760 independent reflections
Radiation source: sealed tube	3490 reflections with *I* > 2σ(*I*)
graphite	*R*_int_ = 0.067
phi and ω scans	θ_max_ = 26.0°, θ_min_ = 1.6°
Absorption correction: multi-scan (*SADABS*; Bruker, 2002)	*h* = −14→15
*T*_min_ = 0.46, *T*_max_ = 0.55	*k* = −22→22
18891 measured reflections	*l* = −13→12

### Refinement

**Table:**

Refinement on *F*^2^	Primary atom site location: structure-invariant direct methods
Least-squares matrix: full	Secondary atom site location: difference Fourier map
*R*[*F*^2^ > 2σ(*F*^2^)] = 0.052	Hydrogen site location: inferred from neighbouring sites
*wR*(*F*^2^) = 0.107	H-atom parameters constrained
*S* = 1.07	*w* = 1/[σ^2^(*F*_o_^2^) + (0.0481*P*)^2^] where *P* = (*F*_o_^2^ + 2*F*_c_^2^)/3
4760 reflections	(Δ/σ)_max_ < 0.001
316 parameters	Δρ_max_ = 0.49 e Å^−^^3^
0 restraints	Δρ_min_ = −0.57 e Å^−^^3^

### Special details

**Table:**

Geometry. All e.s.d.'s (except the e.s.d. in the dihedral angle between two l.s. planes) are estimated using the full covariance matrix. The cell e.s.d.'s are taken into account individually in the estimation of e.s.d.'s in distances, angles and torsion angles; correlations between e.s.d.'s in cell parameters are only used when they are defined by crystal symmetry. An approximate (isotropic) treatment of cell e.s.d.'s is used for estimating e.s.d.'s involving l.s. planes.
Refinement. Refinement of *F*^2^ against ALL reflections. The weighted *R*-factor *wR* and goodness of fit *S* are based on *F*^2^, conventional *R*-factors *R* are based on *F*, with *F* set to zero for negative *F*^2^. The threshold expression of *F*^2^ > σ(*F*^2^) is used only for calculating *R*-factors(gt) *etc*. and is not relevant to the choice of reflections for refinement. *R*-factors based on *F*^2^ are statistically about twice as large as those based on *F*, and *R*- factors based on ALL data will be even larger.

### Fractional atomic coordinates and isotropic or equivalent isotropic displacement parameters (Å^2^)

**Table:**

	*x*	*y*	*z*	*U*_iso_*/*U*_eq_	Occ. (<1)
C1	0.7345 (4)	−0.1231 (2)	0.3081 (4)	0.0385 (10)	
H1	0.6595	−0.1259	0.3029	0.046*	
C2	0.7916 (4)	−0.1886 (2)	0.3052 (4)	0.0347 (10)	
H2	0.7570	−0.2353	0.2975	0.042*	
C3	0.9033 (4)	−0.1825 (3)	0.3155 (4)	0.0394 (10)	
H3	0.9455	−0.2258	0.3174	0.047*	
C4	0.9530 (4)	−0.1133 (3)	0.3213 (4)	0.0419 (11)	
H4	1.0277	−0.1089	0.3257	0.050*	
C5	0.8893 (3)	−0.0504 (3)	0.3225 (5)	0.0391 (10)	
C6	0.9318 (3)	0.0236 (3)	0.3317 (4)	0.0384 (10)	
C7	1.0374 (4)	0.0395 (3)	0.3333 (5)	0.0452 (11)	
H7	1.0858	0.0014	0.3238	0.054*	
C8	1.0714 (3)	0.1123 (2)	0.3497 (4)	0.0348 (10)	
H8	1.1439	0.1241	0.3504	0.042*	
C9	1.0006 (3)	0.1689 (3)	0.3651 (4)	0.0354 (10)	
H9	1.0240	0.2185	0.3790	0.042*	
C10	0.8930 (3)	0.1483 (2)	0.3587 (4)	0.0281 (8)	
H10	0.8423	0.1853	0.3663	0.034*	
C11	0.7170 (3)	0.0426 (2)	0.4978 (4)	0.0363 (10)	
C12	0.5697 (3)	0.0028 (2)	0.2858 (4)	0.0363 (10)	
C13	0.6951 (3)	0.0482 (2)	0.1340 (4)	0.0298 (9)	
C14	0.6443 (3)	0.1349 (3)	0.3103 (4)	0.0385 (10)	
C15	0.8118 (4)	−0.0835 (3)	0.9317 (4)	0.0427 (11)	
H15A	0.8576	−0.0470	0.9859	0.064*	
H15B	0.8564	−0.1163	0.8933	0.064*	
H15C	0.7734	−0.1124	0.9840	0.064*	
C16	0.7459 (4)	0.2236 (3)	0.6177 (4)	0.0390 (10)	
H16A	0.8181	0.2152	0.6712	0.059*	
H16B	0.7319	0.2767	0.6094	0.059*	
H16C	0.7408	0.2022	0.5326	0.059*	
C17	0.4893 (4)	0.1450 (3)	0.9517 (5)	0.0429 (11)	
H17A	0.4907	0.0916	0.9660	0.064*	
H17B	0.4155	0.1608	0.9139	0.064*	
H17C	0.5158	0.1704	1.0336	0.064*	
C18	0.4471 (3)	0.1056 (3)	0.4540 (4)	0.0385 (10)	
H18A	0.4135	0.1343	0.5089	0.058*	
H18B	0.4307	0.0544	0.4617	0.058*	
H18C	0.5235	0.1125	0.4785	0.058*	
Fe1	0.70769 (5)	0.04235 (3)	0.31568 (6)	0.03166 (16)	
N1	0.7825 (3)	−0.05414 (19)	0.3170 (4)	0.0338 (8)	
N2	0.8605 (3)	0.07728 (19)	0.3405 (3)	0.0307 (7)	
N3	0.7168 (3)	0.0475 (2)	0.6043 (4)	0.0364 (8)	
N4	0.4832 (3)	−0.0227 (2)	0.2667 (4)	0.0394 (9)	
N5	0.6896 (3)	0.05129 (18)	0.0266 (4)	0.0339 (8)	
N6	0.6040 (3)	0.1931 (2)	0.3041 (4)	0.0385 (9)	
N7	0.8853 (3)	0.1436 (2)	0.9291 (3)	0.0354 (8)	
O1	0.8747 (2)	0.08407 (17)	0.8686 (3)	0.0398 (7)	
O2	0.7993 (2)	0.17623 (15)	0.9284 (3)	0.0367 (7)	
O3	0.9709 (2)	0.16875 (16)	0.9851 (3)	0.0399 (7)	
O4	0.7341 (2)	−0.04552 (16)	0.8312 (3)	0.0361 (7)	
H4A	0.6659	−0.0724	0.8253	0.054*	
O5	0.6676 (2)	0.18859 (16)	0.6775 (3)	0.0379 (7)	
H5A	0.6452	0.2280	0.7297	0.057*	
O6	0.5579 (2)	0.16346 (17)	0.8642 (3)	0.0354 (7)	
H6A	0.5993	0.2077	0.9002	0.053*	
O7	0.4061 (2)	0.12975 (17)	0.3204 (3)	0.0408 (7)	
H7B	0.4582	0.1451	0.2907	0.061*	
O8	0.3088 (5)	0.2478 (4)	0.4772 (7)	0.0489 (17)	0.50
H8B	0.2447	0.2322	0.4670	0.073*	0.50
H8C	0.3318	0.2622	0.5540	0.073*	0.50
Y1	0.68339 (3)	0.08045 (2)	0.80828 (4)	0.03110 (12)	

### Atomic displacement parameters (Å^2^)

**Table:**

	*U*^11^	*U*^22^	*U*^33^	*U*^12^	*U*^13^	*U*^23^
C1	0.040 (2)	0.028 (2)	0.045 (3)	−0.0085 (19)	0.005 (2)	0.0008 (19)
C2	0.044 (2)	0.033 (2)	0.028 (2)	−0.0147 (19)	0.0103 (18)	−0.0087 (17)
C3	0.039 (2)	0.037 (2)	0.042 (3)	−0.0070 (19)	0.0076 (19)	−0.003 (2)
C4	0.051 (3)	0.035 (2)	0.043 (3)	−0.009 (2)	0.017 (2)	−0.002 (2)
C5	0.030 (2)	0.043 (3)	0.046 (3)	0.0014 (19)	0.0119 (19)	0.001 (2)
C6	0.029 (2)	0.048 (3)	0.039 (3)	−0.0038 (19)	0.0103 (18)	−0.003 (2)
C7	0.041 (3)	0.050 (3)	0.044 (3)	−0.003 (2)	0.008 (2)	0.002 (2)
C8	0.036 (2)	0.038 (2)	0.036 (2)	−0.0024 (18)	0.0191 (18)	0.0030 (19)
C9	0.036 (2)	0.043 (3)	0.029 (2)	0.0085 (19)	0.0124 (17)	−0.0080 (19)
C10	0.034 (2)	0.027 (2)	0.0260 (19)	0.0081 (16)	0.0131 (16)	0.0043 (16)
C11	0.036 (2)	0.033 (2)	0.038 (3)	−0.0010 (19)	0.0049 (19)	0.001 (2)
C12	0.028 (2)	0.031 (2)	0.045 (3)	−0.0038 (18)	−0.0014 (19)	−0.0016 (19)
C13	0.027 (2)	0.029 (2)	0.032 (2)	−0.0095 (16)	0.0043 (17)	0.0037 (17)
C14	0.033 (2)	0.042 (3)	0.046 (3)	0.0122 (19)	0.0193 (19)	−0.002 (2)
C15	0.034 (2)	0.044 (3)	0.040 (3)	−0.013 (2)	−0.0127 (19)	−0.001 (2)
C16	0.037 (2)	0.041 (3)	0.042 (3)	0.003 (2)	0.015 (2)	−0.010 (2)
C17	0.041 (3)	0.038 (3)	0.054 (3)	0.011 (2)	0.018 (2)	0.019 (2)
C18	0.034 (2)	0.037 (2)	0.044 (3)	−0.0078 (18)	0.009 (2)	0.008 (2)
Fe1	0.0315 (3)	0.0311 (3)	0.0327 (3)	−0.0019 (2)	0.0080 (2)	−0.0011 (2)
N1	0.0317 (19)	0.0258 (18)	0.045 (2)	−0.0048 (14)	0.0115 (16)	−0.0053 (15)
N2	0.0281 (17)	0.0279 (18)	0.0366 (19)	−0.0061 (14)	0.0088 (14)	0.0024 (15)
N3	0.0317 (19)	0.041 (2)	0.040 (2)	−0.0017 (15)	0.0149 (16)	0.0024 (17)
N4	0.037 (2)	0.035 (2)	0.045 (2)	0.0037 (16)	0.0059 (17)	−0.0022 (17)
N5	0.0308 (19)	0.0293 (19)	0.043 (2)	−0.0113 (15)	0.0113 (16)	0.0014 (16)
N6	0.036 (2)	0.034 (2)	0.047 (2)	0.0065 (16)	0.0133 (17)	−0.0072 (17)
N7	0.0316 (19)	0.044 (2)	0.0310 (19)	0.0007 (16)	0.0075 (15)	−0.0157 (17)
O1	0.0346 (16)	0.0331 (17)	0.0493 (19)	0.0044 (13)	0.0050 (14)	−0.0012 (14)
O2	0.0293 (16)	0.0297 (16)	0.0476 (19)	−0.0013 (13)	0.0014 (13)	0.0034 (13)
O3	0.0343 (17)	0.0299 (16)	0.0509 (19)	−0.0084 (13)	0.0004 (14)	0.0013 (14)
O4	0.0432 (17)	0.0295 (15)	0.0309 (16)	−0.0158 (13)	−0.0010 (13)	−0.0060 (12)
O5	0.0487 (18)	0.0341 (17)	0.0327 (16)	−0.0156 (14)	0.0128 (13)	−0.0033 (13)
O6	0.0306 (16)	0.0370 (17)	0.0412 (17)	−0.0026 (12)	0.0137 (13)	0.0023 (13)
O7	0.0412 (18)	0.0439 (19)	0.0346 (17)	−0.0068 (14)	0.0034 (13)	0.0051 (14)
O8	0.041 (4)	0.045 (4)	0.058 (4)	0.012 (3)	0.006 (3)	−0.001 (3)
Y1	0.0295 (2)	0.0324 (2)	0.0303 (2)	−0.00059 (17)	0.00460 (15)	0.00014 (17)

### Geometric parameters (Å, °)

**Table:**

C1—N1	1.386 (5)	C16—H16A	0.9799
C1—C2	1.399 (6)	C16—H16B	0.9800
C1—H1	0.9500	C16—H16C	0.9800
C2—C3	1.413 (6)	C17—O6	1.465 (5)
C2—H2	0.9500	C17—H17A	0.9799
C3—C4	1.402 (6)	C17—H17B	0.9800
C3—H3	0.9500	C17—H17C	0.9799
C4—C5	1.404 (6)	C18—O7	1.474 (5)
C4—H4	0.9502	C18—H18A	0.9599
C5—N1	1.356 (6)	C18—H18B	0.9600
C5—C6	1.442 (6)	C18—H18C	0.9600
C6—N2	1.353 (6)	Fe1—N1	1.993 (4)
C6—C7	1.379 (6)	Fe1—N2	2.015 (3)
C7—C8	1.387 (7)	N3—Y1	2.401 (4)
C7—H7	0.9500	N4—Y1^i^	2.344 (4)
C8—C9	1.405 (6)	N5—Y1^ii^	2.384 (4)
C8—H8	0.9500	N7—O3	1.210 (4)
C9—C10	1.413 (6)	N7—O2	1.248 (4)
C9—H9	0.9500	N7—O1	1.251 (4)
C10—N2	1.353 (5)	N7—Y1	2.849 (4)
C10—H10	0.9500	O1—Y1	2.384 (3)
C11—N3	1.146 (6)	O2—Y1	2.448 (3)
C11—Fe1	1.930 (5)	O4—Y1	2.372 (3)
C12—N4	1.173 (5)	O4—H4A	0.9900
C12—Fe1	1.865 (4)	O5—Y1	2.392 (3)
C13—N5	1.140 (5)	O5—H5A	0.9900
C13—Fe1	1.921 (4)	O6—Y1	2.378 (3)
C14—N6	1.169 (5)	O6—H6A	0.9901
C14—Fe1	1.860 (4)	O7—H7B	0.8501
C15—O4	1.459 (5)	O8—H8B	0.8499
C15—H15A	0.9801	O8—H8C	0.8500
C15—H15B	0.9800	Y1—N4^i^	2.344 (4)
C15—H15C	0.9800	Y1—N5^iii^	2.384 (4)
C16—O5	1.455 (5)		
			
N1—C1—C2	122.8 (4)	C12—Fe1—N2	175.02 (17)
N1—C1—H1	118.7	C13—Fe1—N2	88.11 (15)
C2—C1—H1	118.5	C11—Fe1—N2	92.05 (16)
C1—C2—C3	117.2 (4)	N1—Fe1—N2	79.90 (14)
C1—C2—H2	121.6	C5—N1—C1	118.3 (4)
C3—C2—H2	121.2	C5—N1—Fe1	115.7 (3)
C4—C3—C2	120.9 (4)	C1—N1—Fe1	125.9 (3)
C4—C3—H3	119.3	C6—N2—C10	120.4 (3)
C2—C3—H3	119.9	C6—N2—Fe1	114.5 (3)
C3—C4—C5	118.0 (4)	C10—N2—Fe1	125.1 (3)
C3—C4—H4	121.3	C11—N3—Y1	165.9 (4)
C5—C4—H4	120.7	C12—N4—Y1^i^	169.3 (4)
N1—C5—C4	122.7 (4)	C13—N5—Y1^ii^	169.9 (3)
N1—C5—C6	114.2 (4)	O3—N7—O2	121.4 (4)
C4—C5—C6	123.1 (4)	O3—N7—O1	124.0 (4)
N2—C6—C7	121.6 (4)	O2—N7—O1	114.6 (3)
N2—C6—C5	115.1 (4)	O3—N7—Y1	177.2 (3)
C7—C6—C5	123.3 (4)	O2—N7—Y1	58.8 (2)
C6—C7—C8	118.6 (5)	O1—N7—Y1	55.87 (19)
C6—C7—H7	120.5	N7—O1—Y1	98.4 (2)
C8—C7—H7	120.9	N7—O2—Y1	95.3 (2)
C7—C8—C9	121.1 (4)	C15—O4—Y1	130.8 (2)
C7—C8—H8	119.5	C15—O4—H4A	104.8
C9—C8—H8	119.3	Y1—O4—H4A	104.6
C8—C9—C10	116.8 (4)	C16—O5—Y1	130.0 (3)
C8—C9—H9	121.5	C16—O5—H5A	104.7
C10—C9—H9	121.7	Y1—O5—H5A	104.8
N2—C10—C9	121.4 (4)	C17—O6—Y1	123.9 (2)
N2—C10—H10	119.5	C17—O6—H6A	106.4
C9—C10—H10	119.1	Y1—O6—H6A	106.2
N3—C11—Fe1	174.3 (4)	C18—O7—H7B	109.2
N4—C12—Fe1	179.4 (4)	H8B—O8—H8C	109.5
N5—C13—Fe1	178.7 (4)	N4^i^—Y1—O4	79.10 (11)
N6—C14—Fe1	178.6 (4)	N4^i^—Y1—O6	75.80 (12)
O4—C15—H15A	109.3	O4—Y1—O6	140.19 (10)
O4—C15—H15B	109.9	N4^i^—Y1—N5^iii^	93.33 (12)
H15A—C15—H15B	109.5	O4—Y1—N5^iii^	74.86 (11)
O4—C15—H15C	109.2	O6—Y1—N5^iii^	76.29 (11)
H15A—C15—H15C	109.5	N4^i^—Y1—O1	154.27 (12)
H15B—C15—H15C	109.5	O4—Y1—O1	76.06 (10)
O5—C16—H16A	109.1	O6—Y1—O1	128.74 (11)
O5—C16—H16B	109.8	N5^iii^—Y1—O1	86.64 (12)
H16A—C16—H16B	109.5	N4^i^—Y1—O5	102.62 (12)
O5—C16—H16C	109.5	O4—Y1—O5	146.51 (11)
H16A—C16—H16C	109.5	O6—Y1—O5	70.07 (10)
H16B—C16—H16C	109.5	N5^iii^—Y1—O5	137.47 (11)
O6—C17—H17A	109.6	O1—Y1—O5	94.49 (11)
O6—C17—H17B	109.4	N4^i^—Y1—N3	85.22 (13)
H17A—C17—H17B	109.5	O4—Y1—N3	75.66 (12)
O6—C17—H17C	109.4	O6—Y1—N3	131.40 (11)
H17A—C17—H17C	109.5	N5^iii^—Y1—N3	150.21 (13)
H17B—C17—H17C	109.5	O1—Y1—N3	82.26 (12)
O7—C18—H18A	109.0	O5—Y1—N3	71.22 (12)
O7—C18—H18B	109.6	N4^i^—Y1—O2	152.81 (12)
H18A—C18—H18B	109.5	O4—Y1—O2	120.72 (10)
O7—C18—H18C	109.8	O6—Y1—O2	77.33 (10)
H18A—C18—H18C	109.5	N5^iii^—Y1—O2	76.24 (11)
H18B—C18—H18C	109.5	O1—Y1—O2	51.58 (10)
C14—Fe1—C12	87.3 (2)	O5—Y1—O2	71.68 (10)
C14—Fe1—C13	89.19 (19)	N3—Y1—O2	116.23 (11)
C12—Fe1—C13	89.64 (18)	N4^i^—Y1—N7	173.07 (12)
C14—Fe1—C11	87.33 (19)	O4—Y1—N7	98.20 (10)
C12—Fe1—C11	90.5 (2)	O6—Y1—N7	103.03 (10)
C13—Fe1—C11	176.51 (19)	N5^iii^—Y1—N7	79.78 (11)
C14—Fe1—N1	176.55 (18)	O1—Y1—N7	25.75 (10)
C12—Fe1—N1	95.62 (17)	O5—Y1—N7	83.18 (11)
C13—Fe1—N1	88.99 (17)	N3—Y1—N7	100.36 (11)
C11—Fe1—N1	94.48 (17)	O2—Y1—N7	25.85 (9)
C14—Fe1—N2	97.11 (18)		

Symmetry codes: (i) −*x*+1, −*y*, −*z*+1; (ii) *x*, *y*, *z*−1; (iii) *x*, *y*, *z*+1.

### Hydrogen-bond geometry (Å, °)

**Table:**

*D*—H···*A*	*D*—H	H···*A*	*D*···*A*	*D*—H···*A*
O5—H5A···N6^iv^	0.99	1.78	2.762 (5)	172
O7—H7B···N6	0.85	2.03	2.823 (5)	154

Symmetry codes: (iv) *x*, −*y*+1/2, *z*+1/2.
